# Carbonyl reductase 1 is a new target to improve the effect of radiotherapy on head and neck squamous cell carcinoma

**DOI:** 10.1186/s13046-018-0942-9

**Published:** 2018-10-30

**Authors:** Miyong Yun, Ae Jin Choi, Young Chan Lee, Munkyoo Kong, Ji-Youn Sung, Sung Soo Kim, Young-Gyu Eun

**Affiliations:** 1 Department of Bioindustry and Bioresource Engineering, College of Life Sciences, Sejong University, Seoul, Republic of Korea; 2 Department of Otolaryngology-Head & Neck Surgery, School of Medicine, Kyung Hee University, #1, Hoegi-dong, Dongdaemun-gu, Seoul, 02774 Republic of Korea; 3 Department of Radiation Oncology, School of Medicine, Kyung Hee University, Seoul, Republic of Korea; 4 Department of Pathology, School of Medicine, Kyung Hee University, Seoul, Republic of Korea; 5 Department of Biochemistry and Molecular Biology, School of Medicine, Kyung Hee University, Seoul, Republic of Korea

**Keywords:** CBR1, Head and neck squamous cell carcinoma, Ionising radiation, Radiosensitivity, ROS

## Abstract

**Background:**

Human carbonyl reductase 1 (CBR1) plays major roles in protecting cells against cellular damage resulting from oxidative stress. Although CBR1-mediated detoxification of oxidative materials increased by stressful conditions including hypoxia, neuronal degenerative disorders, and other circumstances generating reactive oxide is well documented, the role of CBR1 under ionising radiation (IR) is still unclear.

**Methods:**

The formalin-fixed and paraffin-embedded tissues of 85 patients with head and neck squamous cell carcinoma (HNSCC) were used to determine if CBR1 expression effects on survival of patients with treatment of radiotherapy. Subsequently colony formation assays and xenograft tumor mouse model was used to verify the relationship between CBR1 expression and radiosensitivity in HNSCC cells. Publicly-available data from The Cancer Genome Atlas (TCGA) was analysed to determine if CBR1 expression affects the survival of patients with HNSCC. To verify CBR1-mediated molecular signalling pathways, cell survival, DNA damage/repair, reactive oxygen species (ROS), cell cycle distribution and mitotic catastrophe in HNSCC cells with modulated CBR1 expression by knockdown or overexpression were measured using by colony formation assays, flow cytometry, qRT-PCR and western blot analysis.

**Results:**

HNSCC patients with low CBR1 had a significantly higher survival rate than the high CBR1 expression (84.2% vs. 57.8%, *p* = 0.0167). Furthermore, HNSCC patients with low CBR1 expression showed a good prognosis for IR compared to patients with highly expressed CBR1. Also, we found that IR upregulated CBR1 mRNA via Nrf2 activation in HNSCC cells and patients. In vitro analysis, we found that CBR1-specific siRNA or inhibitor significantly enhanced radiosensitivity after IR, while CBR1 overexpression decreased. CBR1 inhibition by siRNA or inhibitor treatment accumulated cellular ROS leading to aberrant DNA damage repair and an increase of mitotic catastrophe. Moreover, the combination of CBR1 depletion with IR dramatically inhibited primary tumour growth in a xenograft tumor mouse model.

**Conclusion:**

Our findings indicate that CBR1 has a key role in DNA damage response through regulation of IR-mediated ROS generation. Consistently, CBR1 expression is highly correlated with patient survival after and susceptibility to radiation therapy. Therefore, CBR1 inhibition with IR might be a potent therapeutic strategy for HNSCC treatment.

**Electronic supplementary material:**

The online version of this article (10.1186/s13046-018-0942-9) contains supplementary material, which is available to authorized users.

## Background

Head and neck squamous cell carcinoma (HNSCC) arising most commonly from the mucosa of the oral cavity, pharynx, and larynx, is the sixth most common cancer, with an incidence rate of approximately 600,000 patients per year worldwide [1]. Despite advances in our knowledge of its epidemiology, pathogenesis, and treatment modalities, the survival rates of HNSCC have not improved over the past four decades, remaining at a 50% 5-year survival rate [2].

Ionising radiation (IR), one of the main treatments for HNSCC, causes damage to DNA, lipids, and proteins due to the energy of the radiation and by reactive oxygen species (ROS), which is derived from intercellular water. Among these effects, ROS induced by IR is critical for the induction of cell death. In the context of the cellular response to radiation, the contributions of the radiation-oxidative damage of the membranes as well as the DNA damage by ROS appear quite complex, and these methods of cytotoxicity seem closely linked in the development of the radiation-induced cell death [3]. While ROS is a critical factor of ionising radiation-mediated tumour cell death, the expression of antioxidants in tumours prevents DNA damage and protects cells from irradiation-mediated cell death [4, 5]. Hence, many therapeutic strategies are designed to excessively increase cellular ROS levels causing tumour cell death via accumulation of irreparable damage [6]. NADPH-dependent, monomeric, and cytosolic enzyme belonging to.

CBR1 is a subfamily enzyme of the short-chain dehydrogenase/reductase. CBR1 inactivates highly reactive lipid aldehydes, such as 4-oxonon-2-enal (ONE), 4 hydroxynon-2-enal (HNE), and acrolein, which are able to modify proteins and are capable of producing DNA damage within cells [7]. Therefore, CBR1 may play a meaningful role for preserving cell from oxidative stress. Overexpression of human CBR1 in NIH3T3 cells provided protection from ROS-induced cellular damage [8]. It was reported that HIF1-α, AP-1, and Nrf2 could regulate CBR1 at the transcriptional level [9–11].

It is not known whether CBR1 affects the results of radiotherapy, but it is likely because IR increases endogenous expression of many antioxidants by acting as oxidative stress. We hypothesised that radiation exposure would activate CBR1 and inhibition of CBR1 activity could enhance radiation sensitivity by decreasing the capacity of CBR1 to scavenge IR-induced ROS.

## Materials and methods

### Chemical

Hydroxy-PP-Me was synthesized following by previous method [12]. Briefly, Hydroxy-PP-Me was synthesized in three cascade steps; 4-chloro-5-iodo-7H-pyrrolo [2,3-d] pyrimidine (compound 1), N-alkylated pyrrolopyrimidine (compound 2) and Hydroxy-PP-Me (compound 3).

### Cell culture

The HNSCC cell lines YD8 and SCC4 were kindly provided by Prof. Kwang Seok Ahn (Kyung Hee University) and SNU-1041 by Prof. Chul Ho Kim (Aju University). FaDu and YD-10B were purchased from Korean cell line bank (Seoul, Korea). YD8, SNU-1041, and YD10B were cultured in RPMI-1640 (Corning, Manassas, VA, USA). FaDu were cultured in MEM (Corning) and SCC4 in DMEM (Corning), supplemented with 10% FBS (Corning) and 1% penicillin-streptomycin (Corning). All cell lines were cultured at 37 °C in the presence of 5% CO_2_.

### Plasmid and siRNA transfection

GFP-conjugated (pCMV6-AC-GFP) empty or CBR1 plasmids were purchased from Origene (Rockville, MD, USA). The siRNAs were purchased from IDT (Cambridge, MA, USA). Cells were transfected with plasmid or siRNA using TransIT-LT1 or TransIT-TKO Transfection Reagent (Mirus Bio, Madison, WI, USA) according to the manufacturer’s recommendations.

### Generation of stable shCBR1 knockdown cells

A set of non-targeting shRNA (shNT) and three shRNA targeting different coding regions of the CBR1 gene (shCBR1) were purchased from Sigma Aldrich Biosciences (shNT; SH001V, shCBR1; TRCN0000046375, TRCN0000046377, TRCN0000289028). FaDu and YD-10B cells were subsequently transduced and selected on 2 to 2.5 μg/ml puromycin to create the stable inducible shRNA line.

### Radiation

Cells were exposed to fresh media containing the inhibitor or relative DMSO control for 3 h prior to irradiation (IR) in tissue culture vessels using an XStrahl RS225 cabinet at room temperature with 195 kV/15 mA X-rays producing a dose rate of 1.6 Gray per minute.

### Luciferase activity assays

Reporter gene constructs comprising CBR1 promoter sequences of 1003-bp upstream of the CBR1 translation initiation codon and empty vector, pGL3-Basic (Promega, Madison, WI), were co-transfected with the pRL-CMV plasmid (Renilla luciferase, Promega) into 70% confluent cell cultures using TransIT-LT1 (Mirus Bio.). The activities of the luciferase reporter gene were determined with the Dual-Luciferase Reporter Assay System (Promega) per the manufacturer’s instructions. Light intensity was measured in a Spectramax luminometer (Molecular Devices, Sunnyvale, CA) equipped with proprietary software for data analysis (Softmax pro; Molecular devices).

### Colonogenic survival assay (CSA)

Exponentially growing cells were transfected with control siRNA oligos or gene-specific siRNAs for 24 h. HNSCC cells were plated in 60-mm dishes and grown for 24 h to 70% confluence before irradiation. HNSCC cells were treated with Hpp-Me for 3 h before irradiation. Cells were irradiated with 2 to 6 Gy. Cells were allowed to grow for 10 to 14 days followed by fixing and staining with Gentiana violet. Colonies containing more than 50 cells were counted. To determine survival fraction, colony-forming efficiency was determined, averaged, and normalised to those of the non-irradiated control. Ionising radiation-dependent cell survival curves were fitted using the linear-quadratic (LQ) model. The significance of the difference between the dose responses was calculated by conducting a two-way ANOVA test.

### Western blotting

After irradiation, cells were lysed in RIPA buffer (50 mM Tris-HCl, 150 mM NaCl, 2 mM EDTA, and 1% TritonX-100) containing protease inhibitors (Roche, Germany) and phosphatase inhibitors (Sigma, USA)) for 10 min on ice. The equal amounts of samples were separated on 8 to 12% SDS-polyacrylamide gel, electrophoresed, and transferred to polyvinylidene difluoride membranes (Millipore, Billerica, MA, USA). The membranes were blocked and probed with primary antibodies for anti-CBR1 (Novus) or anti-α-tubulin (Abcam, Cambridge, MA, USA), and incubated with horseradish peroxidase-conjugated secondary antibodies (Cell Signaling, Beverly, MA, USA).

### Real-time quantitative reverse transcriptase PCR analysis

Total RNA was extracted from the indicated cell lines using an RNeasy mini kit (Qiagen, Hilden, Germany) according to the manufacturer’s instructions. RNA was reverse transcribed to cDNA with qPCRBIO cDNA Synthesis kit (PCR BIOSYSTEMS. London, UK). The resulting cDNA was assayed by using real-time quantitative RT-PCR with 2X qPCRBIO Sybr Green Mix (PCR BIOSYSTEMS). Primers for the endogenous CBR1, GAPDH were CBR1, 5′-CAGAGACCCCTGTGTACTTG-3′ (sense); CBR1, 5′-CAACTCAGGACAAGGTACAAAATG-3′ (antisense). Relative amounts of mRNA were calculated from the threshold cycle number using expression of GAPDH as an endogenous control. All experiments were performed in triplicate and the values averaged.

### γ-H2AX immunofluorescence staining

DNA double-strand break (DSB) kinetics were studied using γ-H2AX foci immunofluorescence staining. Cells were plated on glass coverslips in a 24-well plate. After treatment, cells were washed with PBS and fixed using 10% formalin for 10 min at room temperature. Next, specimens were blocked with the supernatant of 5% BSA/PBS, stirred for 1 h, and incubated with a rabbit-polyclonal antibody against γ-H2AX (phosphor S139, Abcam) overnight at 4 °C followed by washes and incubation with Alexa-488-labelled anti-rabbit secondary antibodies (Abcam) for 1 h at room temperature. Specimens were counter stained with 4′,6-diamidino-2-phenylindole (DAPI) and washed in 0.1% PBS-Tween20 before mounting with Vectashield mounting medium (Vector Labs, Inc. Burlingame, CA, USA). Fluorescent images were obtained with a confocal microscope (Zeiss LSM710, Germany).

### Detection of ROS production

Exponentially growing cells were transfected with control siRNA oligos or gene-specific siRNAs for 24 h. Cells were irradiated with 6 Gy using a 250-kVp x-ray (0.61 Gy/min). Twenty-four hours after IR, the level of intracellular ROS was monitored using the total ROS detection kit according to the manufacturer instructions (Enzo life sciences, Farmingdale, NY, USA).

### Xenograft mouse model

Male BALB/c nude mice, weighing 20 ± 2 g were purchased from the Orient (Seongnam, Korea). This study was approved by and conducted in accordance with the policies set forth Kyung Hee Medical Center Institutional Animal Care and Use Committee (KHMC-IACUC-16-039). Stable shCBR1 knockdown cells (2.5 × 10^6^ cells per mouse in 0.1 ml saline) were injected subcutaneously in the right thigh of each mouse, and the tumours were allowed to grow. When the volume of tumours had reached 70–100 mm^3^, the mice were divided randomly into different groups of 5 mice per group. The animals were weighed weekly. The longest and shortest lengths (d_long_ and d_short_) were measured twice a week at right angles for defining the diameter of the tumour with electronic callipers and converted to volume by the formula volume = [(d_short_)^2^ × (d_long_)]/2. The following radiation schedule was used during this study: 2 Gy daily for 5 days (10 Gy total). The 5 × 2 Gy-fractionated IR dose was used to mimic the protocol classically applied to a patient for a week of treatment.

### HNSCC tissue samples and immunohistochemistry

The formalin-fixed and paraffin-embedded 85 HNSCC tissues were used in this study. All patients received definite radiotherapy or adjuvant radiotherapy. The study was approved by the institutional review board (IRB) of Kyung Hee University Medical Center (KMC IRB- 1444-04). Immunohistochemistry (IHC) was carried out on 4-μm tissue sections using the Bond Polymer Refine Detection System (see Supplementary Methods for tumour sections).

### Immunohistochemistry interpretation and analysis

All sections were examined by an expert pathologist who was blinded to the clinical data. Cytoplasmic staining was considered positive for CBR1. Staining intensity was defined as follows: 0, no staining; 1, weak; 2, moderate; and 3 strong. Quantification of positivity (0–100%) was based on an estimate of the percentage of tumour cells at each intensity (0–3). The final immunoreactivity score was obtained by the following equation: final immunoreactivity score = (proportion of tumour cells with no staining × 0) + (proportion of tumour cells with weak intensity × 1) + (proportion of tumour cells with moderate intensity × 2) + (proportion of tumour cells with strong intensity × 3), resulting in immunoreactivity scores ranging from 0 to 300. The cut-off value was calculated by time-dependent ROC curve analysis. An immunoreactivity score that was higher than the cut-off value was considered high expression.

### Statistical analysis

All experiments were performed more than 3 times. Data were processed and analysed using the R language environment (http://www.r-project.org). Independent *t*-testing was used to compare the values between 2 groups. We estimated prognoses using Kaplan-Meier analysis and the log-rank test. *P* value less than 0.05 indicated statistical significance.

## Results

### HNSCC patients with low CBR1 expression show a good prognosis for radiation therapy

**Fig. 1:**
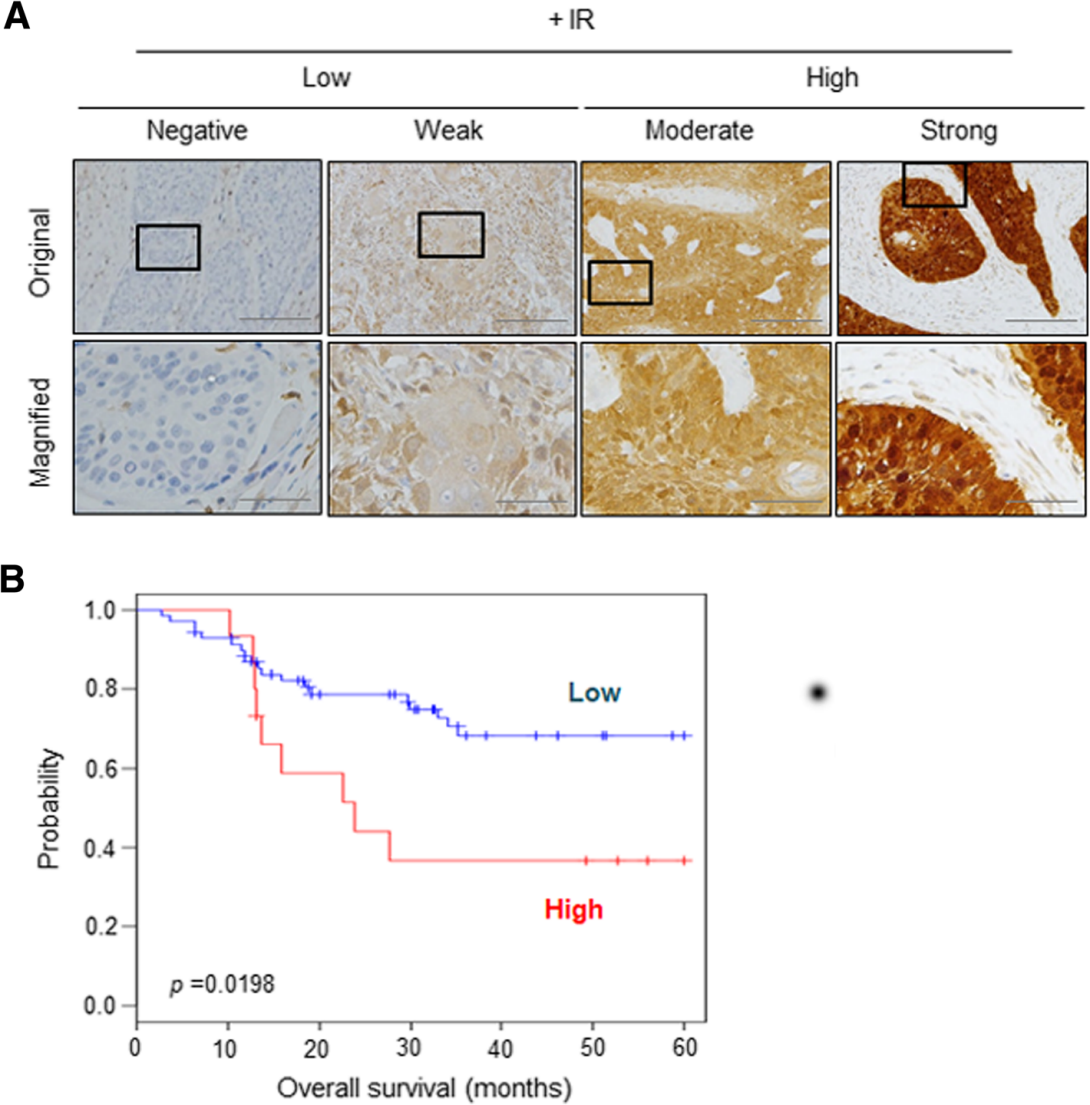
HNSCC patients with low CBR1 expression show a good prognosis for radiation therapy. **a**, Immunohistochemical analysis. Typical staining intensities from no staining to strong staining are shown, using tissues of HNSCC patients who received radiation therapy. **b**, Kaplan-Meier curves for overall survival based on immunoreactivity score in HNSCC patients that received radiotherapy (*n* = 85). HNSCC patients were classified into the patients with low CBR1 (*n* = 70) and the patients with high CBR1 (*n* = 15). Scale bar of non-magnified; 200 μm, magnified; 50 μm

To verify whether CBR1 is a prognostic factor for HNSCC patients, we analysed its expression in cohorts of the publicly available database (https://www.ncbi.nlm.nih.gov/geo/). One hundred seventy-four HNSCC patients were enrolled from GSE42743, GSE10300, and GSE25727 [13–15]. Additional file 1: Table S1 shows the pathological and clinical characteristics of the patients in all three cohorts. These patients were divided into high and low groups based on the median value of CBR1. The low CBR1 group had a significantly higher survival rate than the high CBR1 group (84.2% vs. 57.8%, *p* = 0.0167) (Additional file 2: Figure S1). It was found that low-expression CBR1 groups had a better disease-free survival rate, although this was not limited to patients receiving radiotherapy because of the limitations of the data. Next, to confirm that CBR1 expression indeed influences the results of radiation treatment, we examined whether CBR1 expression is the prognostic factor in 85 patients with head and neck cancer who were treated with radiation therapy (Table 1). We used the immunoreactivity score to investigate both the staining intensity and quantification of the IHC (Fig. 1a). The 5-year overall survival (OS) rate of patients with high CBR1 expression was 40%, and that of patients with low CBR1 expression was 72.9%, meaning that the prognosis of patients with low CBR1 expression was significantly better (*p* = 0.0198, Fig. 1b). The immunoreactivity score of CBR1 in patient tissues showed the possibility of an indicator for the prognosis of effectiveness of radiation therapy.

**Table 1:** Patient’s characteristics (*n* = 85)

Characteristics	Number
Gender	
Male	64 (73.7%)
Female	21 (26.3%)
Age (mean ± SD)	62.0941
Anatomic site	
Oral cavity	27 (31.2%)
Oropharynx	16 (19.0%)
Larynx	19 (22.5%)
Hypopharynx	15 (17.8%)
others	8 (9.5%)
Primary tumor	
T1	19 (22.9%)
T2	22 (26.5%)
T3	16 (19.3%)
T4	26 (31.3%)
Regional lymph node	
N0	36 (42.4%)
N1	13 (15.3%)
N2	35 (41.2%)
N3	1 (0.01%)
Stage	
I	11 (13.1%)
II	14 (16.7%)
III	10 (11.9%)
IV	49 (58.3%)
Tobacco use	
Never	35 (41.1%)
Yes	50 (58.9%)

### Inhibition of CBR1 increases radiosensitivity

**Fig. 2:**
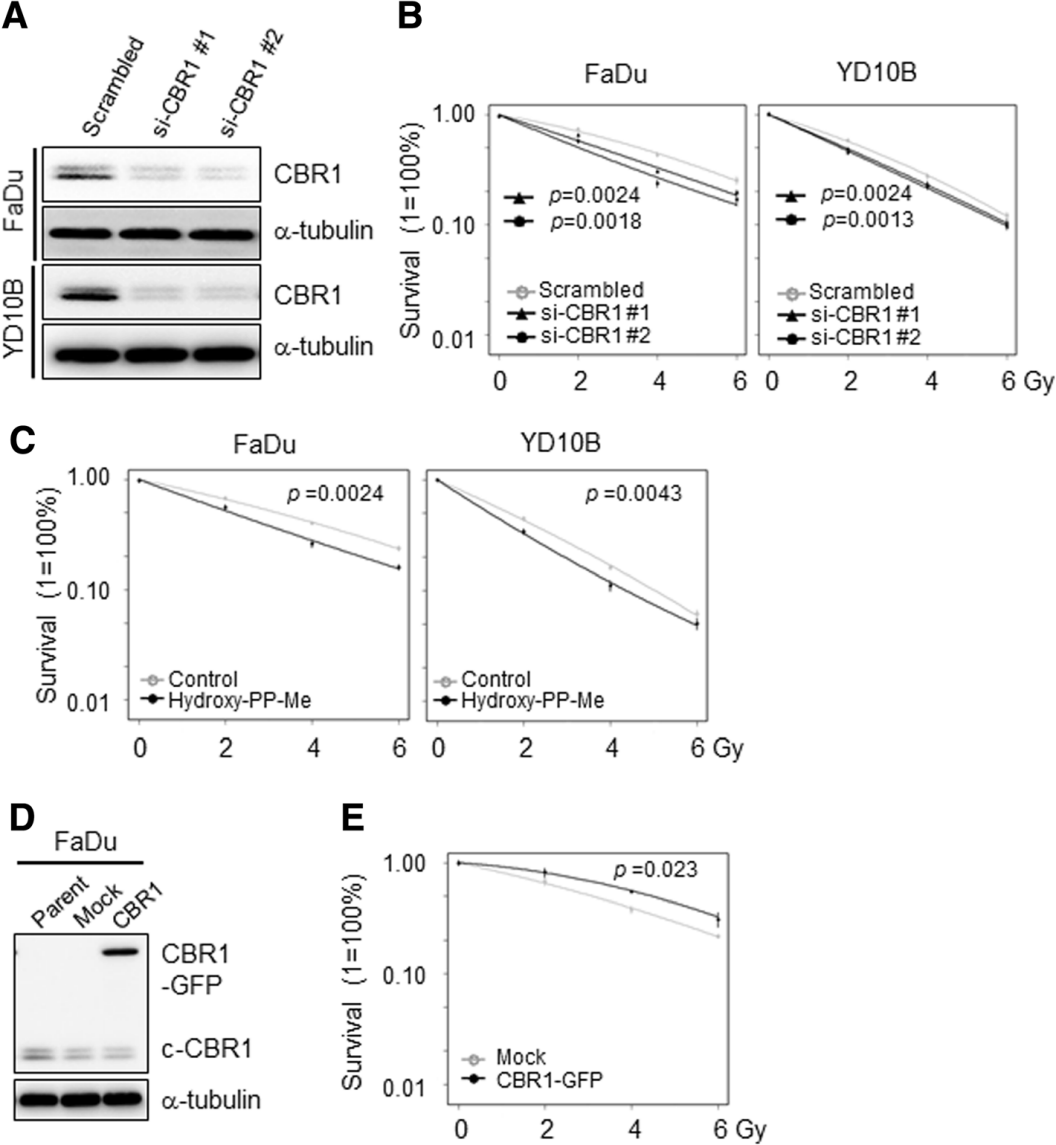
CBR1 participates in radiation sensitivity of HNSCC cells. **a**, CBR1 protein expression was evaluated in FaDu and YD10B cells transfected with scrambled or CBR1-specific siRNA. **b**, Colony forming efficiency of cells transfected for 24 h with scrambled or CBR1 siRNA, prior to IR. Radiation sensitivity was increased by CBR1 knockdown (two-way ANOVA; *n* = 4; *, *P* < 0.05; **, *P* < 0.01; ***, *P* < 0.001 vs. Scrambled siRNA). **c**, Colony forming efficiency of cells treated with 25 μM hydroxy-PP-Me (CBR1 inhibitor) before and after IR. CBR1 inhibitor significantly decreased cell survival with IR (two-way ANOVA; *n* = 4; *, *P* < 0.05; **, *P* < 0.01; ***, *P* < 0.001 vs. control). **d**, CBR1 protein expression was evaluated in FaDu and YD38 cells transfected with Mock and CBR1/WT vectors. **e**, Colony forming efficiency of cells transfected for 24 h with Mock or CBR1/WT vectors, prior to IR. Radiation sensitivity was reduced by CBR1 overexpression (two-way ANOVA; *n* = 4; *, *P* < 0.05; **, *P* < 0.01; ***, *P* < 0.001 vs. Mock)

To explore the role of CBR1 in radiation sensitivity, we modulated CBR1 expression in HNSCC cells by transfection with specific siRNA or overexpression plasmid (Fig. 2a and d). In the radiation sensitivity assay, we found that CBR1 inhibition by siRNA transfection significantly decreased surviving fraction at all IR doses compared with scramble in FaDu and YD10B cells (Fig. 2b, Additional file 2: Figure S2A). Consistently, treatment of 3-(7-isopropyl-4-(methylamino)-7H-pyrrolo [2,3-d] pyrimidin-5yl) phenol (hydroxy-PP-Me), a specific inhibitor of CBR1 [12], also significantly reduced cell survival at all IR doses in FaDu and YD10B cells (Fig. 2c and Additional file 2: Figure S2B) meaning increase of radiosensitivity. Next, to confirm whether CBR1 overexpression inversely affects cell survival, we constructed the stable cell lines with CBR1 expression plasmid. When these cells were treated with IR, cells overexpressing CBR1 exhibited greater survival than mock-transfected cells (Fig. 2e and Additional file 2: Figure S2C) indicating resistance to IR. Taken together, these results strongly indicate that CBR1 participates in radiation sensitivity in HNSCC cells.

### CBR1 inhibition increases intracellular ROS leading to accumulation of DNA damage and induction of mitotic catastrophe

**Fig. 3:**
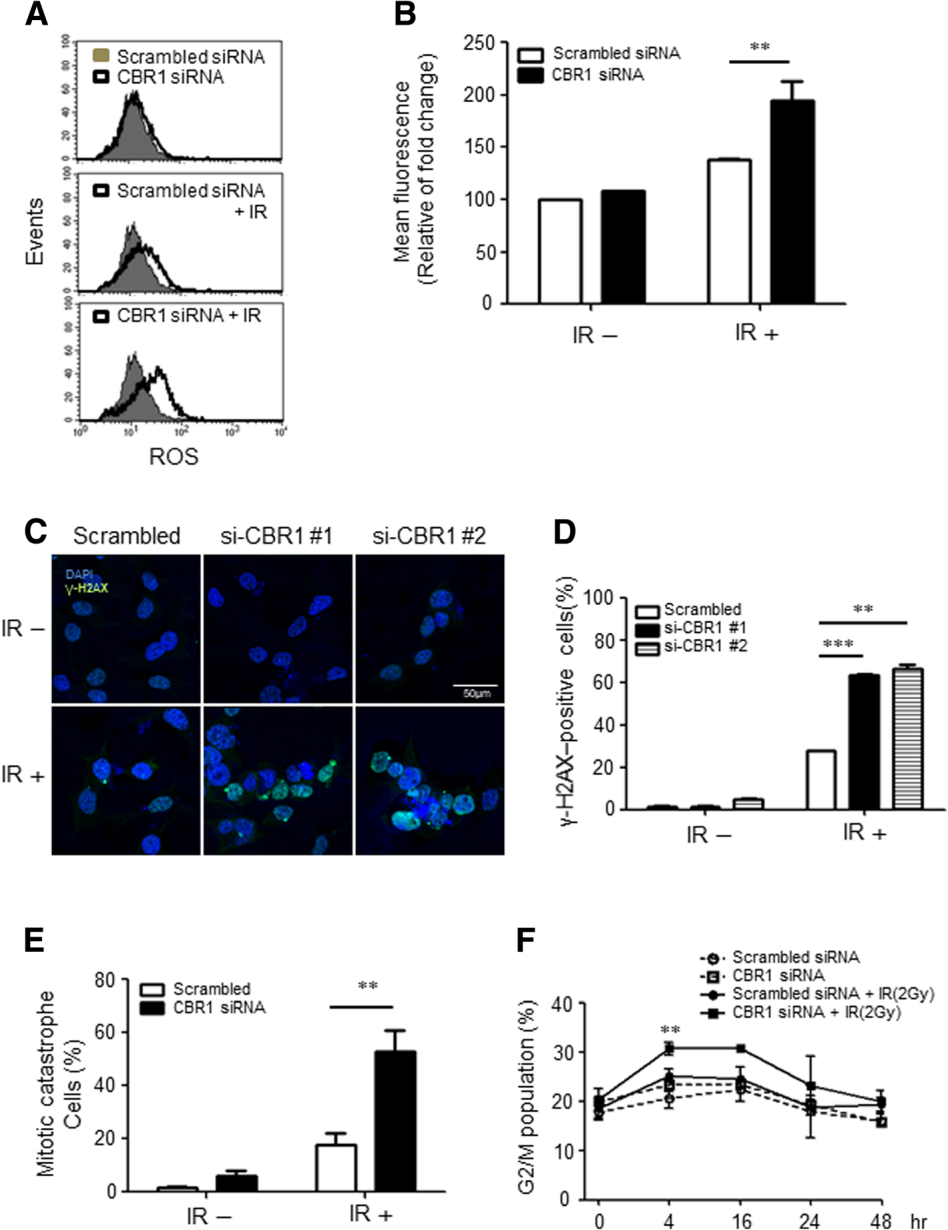
Depletion of CBR1 accumulates ROS, leading to increased DNA damage and mitotic catastrophe. **a**, Scrambled siRNA and CBR1-siRNA were transfected into FaDu cells and then exposed to 6 Gy IR. ROS formation was analysed by FACS using a total ROS detection kit (Enzo) 24 h after IR. **b**, Mean fluorescence from three independent experiments (two-way ANOVA; **, *P* < 0.01 vs. Scrambled siRNA). **c** and **d**, Detection of γH2AX foci formation was performed 48 h after IR (6 Gy) with scrambled siRNA or CBR1 siRNA pre-treatment prior to IR. B, Error bar summarising immunofluorescent data from analysis of ~ 200 cells (two-way ANOVA; ***, *P* < 0.001 vs. Scrambled siRNA). **e**, Representative image and summary of the percentage of FaDu cells with micronuclei (arrows) 48 h following treatment with IR. Error bars show mean average from scoring ~ 200 nuclei (*t*-test; **, *P* < 0.01). **f**, FaDu cells were transfected with scrambled or CBR1 siRNA for 24 h and then irradiated with 2 Gy. Cells were collected at each time point thereafter and analysed by flow cytometry for the percentage of cells in G2/M (two-way ANOVA; **, *P* < 0.01 vs. Scrambled siRNA)

Previous clinical and in vitro data clearly show that CBR1 plays a critical role in radiation sensitivity. To ascertain the role of CBR1 function in radiation sensitivity, we measured intracellular ROS levels under CBR1 depletion in HNSCC cells. Fig. 3a and b show that CBR1 depletion meaningfully increases the ROS level by 89% and 61% compared to the siRNA control only and siRNA control plus IR, respectively (Fig. 3a and b). The consequence of aberrant DNA repair or accumulated damage beyond that of repair capacity after IR is the onset of cell death such as apoptosis, mitotic catastrophe, or senescence [16]. To elucidate whether increases in ROS by CBR1 depletion can exacerbate DNA damage, we measured the profile of DNA damage by immunofluorescence assay using phosphorylated histone 2AX foci (γH2AX). After IR, the γH2AX-positive cells were significantly increased in CBR1-siRNA transfected cells compared to cells with scrambled-siRNA (*p* = 0.0029 and *p* = 0.0004) (Fig. 3c and d). This suggests that the ROS accumulation by inhibition of CBR1 exacerbates DNA damage. Next, to determine whether the increase in radiosensitivity resulting from CBR1 inhibition was due to an enhancement of radiation-induced apoptosis, we examined the apoptotic protein after IR treatment in CBR1-siRNA and scrambled-siRNA transfected cells. There was no significant difference in apoptotic protein expression between the two cells (Additional file 2: Figure S3). Given the apparent DNA repair and lack of increase in radiation-induced apoptosis after CBR1 inhibition, we hypothesised that the mechanism of cell death involved an increase in radiation-induced mitotic catastrophe. Cells in mitotic catastrophe can be defined as having nuclear fragmentation that can be defined as two or more distinct nuclear lobes within a single cell. As shown in Fig. 3e and Additional file 2: Figure S4, CBR1 inhibition resulted in a significant increase in the percentage of cells in mitotic catastrophe after IR. Several anticancer drugs are known to be able to induce mitotic catastrophe and G2/M arrest simultaneously in cancer. Next, we investigated whether CBR1 can enhance radiation-induced mitotic arrest. Cell cycle analysis using flow cytometry of PI-stained cells revealed that CBR1 inhibition followed by radiation resulted in significant cell cycle arrest in the G2-M phase across the cell lines tested, compared with either control cells or IR alone (Fig. 3f and Additional file 2: Figure S5). These results suggest that the increase in radiosensitivity following CBR1 inhibition results from accumulation of ROS and DNA damage, which then contributes to an increase in the number of cells undergoing mitotic catastrophe and mitotic arrest in HNSCC cells.

### Ionising radiation transcriptionally increases CBR1 expression in HNSCC cells

**Fig. 4:**
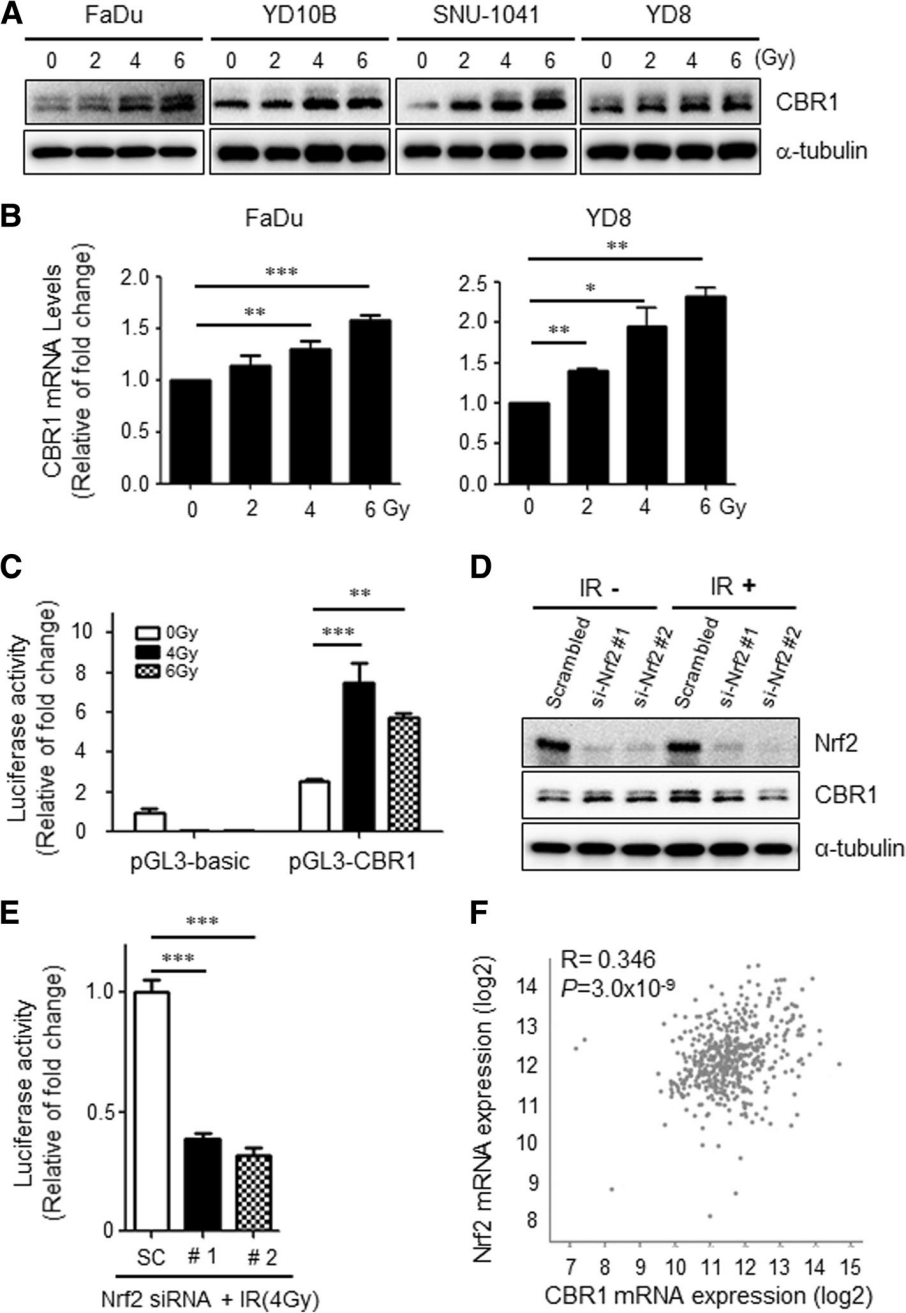
Ionising radiation increases CBR1 mRNA via Nrf2 activation in HNSCC. **a**, CBR1 expression by western blot analysis 24 h after IR from 0 to 6 Gy in HNSCC cells (FaDu, YD10B, SNU-1041, YD8). α-tubulin was used as an internal loading control. **b**, Total RNA extracted from FaDu and YD8 cells measured by quantitative RT-PCR analysis (*t*-test; *n* = 4; *, *p* < 0.05; **, *P* < 0.01; ***, *P* < 0.001). **c**, FaDu cells were transfected with the luciferase reporter constructs prior to IR. Luciferase activity was assayed 48 h after IR. The relative luciferase activities are expressed in comparison with the activity of the pGL3-Basic construct (two-way ANOVA; **, *P* < 0.01; ***, *P* < 0.001). **d**, FaDu cells were transfected with scrambled or Nrf2 siRNA, prior to IR. Nrf2 and CBR1 expression levels were measured 48 h after IR (4 Gy). **e**, FaDu cells were transfected with each CBR1 reporter and treated with Scrambled siRNA or Nrf2-siRNA prior to IR. Luciferase activity was examined 48 h after IR (4 Gy, right panel) (*t*-test; *n* = 3; ***, *P* < 0.001 vs. Scrambled siRNA). **f**, The correlation was evaluated between Nrf2 mRNA expression and CBR1 mRNA expression from TCGA data (sample number = 279)

Based on previous reports indicating that CBR1 expression is regulated in stress conditions, we sought to determine if exposure to varying doses of IR would activate the CBR1 [9, 11]. A dose-dependent increase in CBR1 protein was observed after IR from 0 to 6 Gy in HNSCC cell lines YD10B, FaDu, SNU-1041, and YD8 (Fig. 4a). To verify whether upregulation of CBR1 protein was occurring from the transcriptional stage, we performed quantitative RT-PCR. The results showed that the level of CBR1 mRNA is also dose-dependently increased after IR (Fig. 4b). Consistently, the promoter reporter assay (Additional file 2: Figure S6) showed that IR dramatically increases the luciferase-activity of the plasmid-containing CBR1 promoter by more than 4-fold compared with the control (Fig. 4c), meaning that CBR1 is transcriptionally upregulated by IR in a variety of HNSCC cells.

### Nrf2 transcriptionally regulates CBR1 expression under IR and is correlated with CBR1 expression in tumour tissues of HNSCC patients

Nrf2 is known to be important to maintain resistance to IR. Thus, we sought to determine whether Nrf2 could regulate CBR1 transcription after IR in HNSCC cell lines. To prove this hypothesis, we conducted a promoter assay using the reporter plasmid containing CBR1 promoter. In Nrf-2 depleted cells (Additional file 2: Figure S7), luciferase activity including that of the CBR1 promoter was dramatically reduced and was 2-fold higher than scramble siRNA. In addition, CBR1 protein was significantly downregulated in cells with Nrf2 knockdown after IR, while Nrf2 depletion did not affect CBR1 protein expression without IR treatment (Fig. 4d and e). Next, we analysed the correlation of these two genes’ expression in HNSCC patients (*n* = 279) using the TCGA database (www.cbioportal.org). Interestingly, this analysis showed that transcriptional expression of CBR1 is moderately correlated with the Nrf2 mRNA level (*r* = 0.346, *p* = 3.0 × 10^− 9^) (Fig. 4f). These results suggest that Nrf2 is a major transcriptional factor to regulate CBR1 mRNA expression after IR in HNSCC cells and has a moderate correlation with CBR1 in vivo.

### CBR1 silencing enhances the radiation-induced regression of tumours in xenograft models

**Fig. 5:**
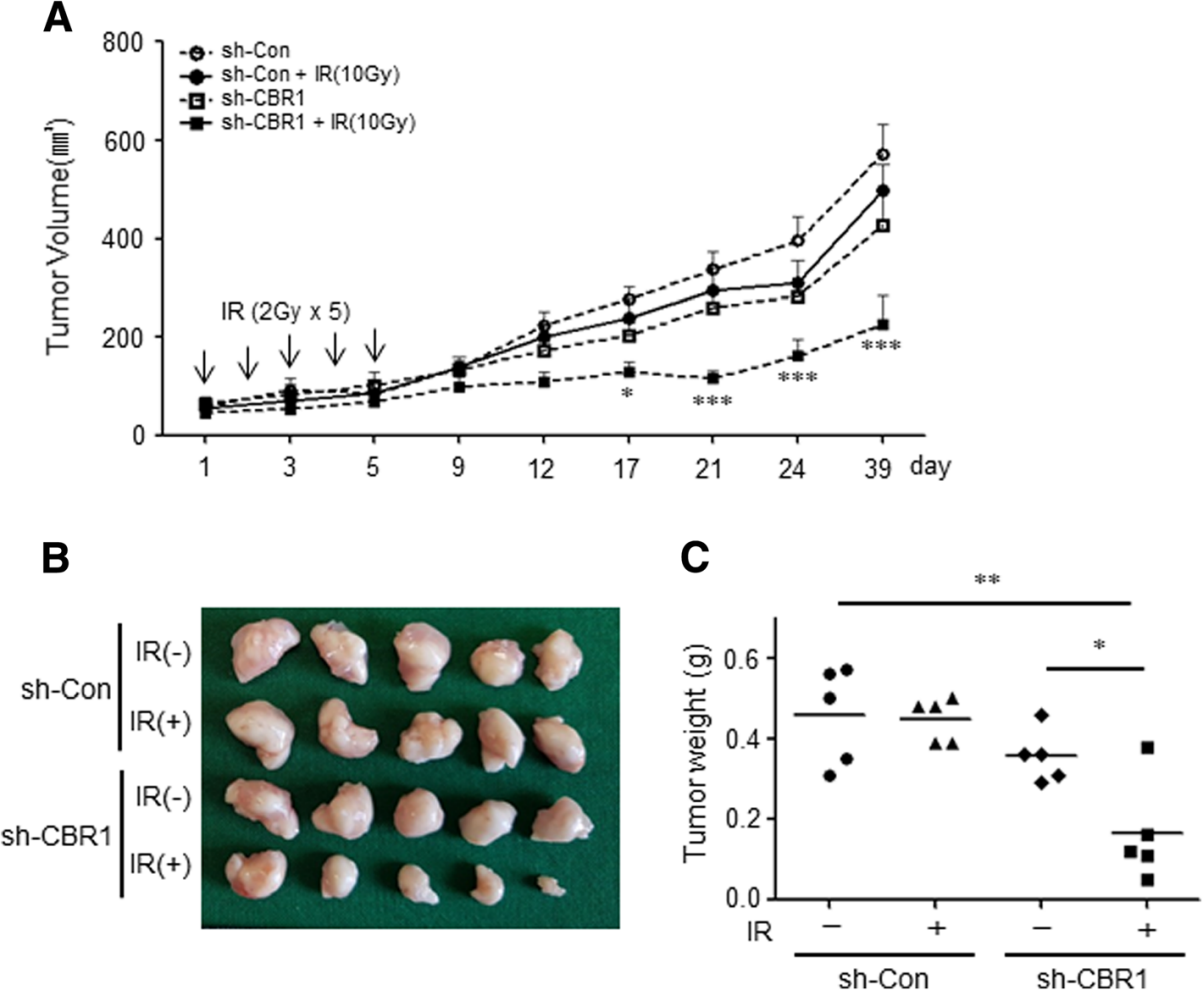
CBR1 inhibition increases radiosensitivity in HNSCC xenograft mouse model. **a**, In YD10B tumour xenograft mice in which radiation treatment (2 Gy) was administered once every other day, the tumour volume reached 70–100 mm^3^ after a total of 5 days. Tumour volume was measured at the indicated days after IR (two-way ANOVA; *n* = 5; *, *p* < 0.05; ***, *P* < 0.001). **b**, Photograph of the dissected tumours. **c**, Tumour weight was measured in the indicated groups (*t*-test; *n* = 5; *, *p* < 0.05; **, *P* < 0.01)

Based on significant in vitro data and patient studies, we conducted a preclinical evaluation in an in vivo model, where YD10B cells were used to generate xenograft tumours in BALB/c Nude mice that were randomised into four treatment groups: vehicle, radiation, shCBR1, and shCBR1 plus radiation. All four treatment regimens were well-tolerated, as total mouse body weights remained unchanged (< 10% fluctuation) throughout the 38-day experiments (data not shown). To determine the combined effect of CBR1 inhibition and IR on tumour growth in vivo, tumour growth was measured for up to 39 days after IR. The tumour volumes in mice treated with both CBR1 inhibition and IR showed a significant difference (smaller 2-fold), compared with the single treatment group with CBR1 shRNA or IR alone from day 12. This difference between the combined and single groups was larger after day 21. At the end of treatment (40 days), the mean tumour volume decreased by 60% compared to the control group (*P* < 0.001) and by 54.7% compared to the irradiated control groups (*P* < 0.001) (Fig. 5a and Additional file 2: Figure S8). In addition, statistically significant differences were found in tumour weight between the combination treatment and the single treatment groups (Fig. 5b and c). The result showed that the combination of CBR1 inhibition and IR significantly suppressed tumour growth compared with vehicle, only radiation, or only CBR1 inhibition. The CBR1 inhibition significantly enhanced the radiation sensitivity of HNSCC in vivo.

## Discussion

In this study, we sought to assess whether radiation exposure would activate CBR1 and if CBR1 inhibition could enhance radiation sensitivity. The findings of this study are as follows: [1] IR increased the activity of CBR1; [2] CBR1 inhibition increased the radiation sensitivity measured by clonogenic assay in vitro; [3] overexpression of CBR1 protected the HNSCC cells against IR; [4] CBR1 inhibition resulted in accumulated intracellular ROS levels leading to an increase in mitotic catastrophe and mitotic arrest; and [5] of the patients treated with radiation, patients with low CBR1 expression showed better prognosis. These results suggest that CBR1 is essential for the survival of cancer cells after IR and can be a good target in developing radiosensitisers.

HNSCC is challenging to treat effectively while maintaining the function of vital healthy structures. Radical surgical resection of the primary tumour and regional cervical lymph nodes used to be the standard of treatment. Concurrent chemoradiotherapy (CCRT) and radiotherapy after radical surgery improves the survival of locally advanced head and cancer patients [17, 18]. More recently, organ-preserving strategies using either radiation alone or CCRT have become a treatment option for HNSCC patients and have been the focus of much investigation. The systematic clinical investigation of organ-preserving radiotherapy and CCRT regimens suggested that these regimens could produce overall survival results as good as surgical resection for patients with advanced HNSCC; thus, radiation has become a cornerstone of treatment for patients with advanced HNSCC [19–22]. Despite advances in radiotherapy techniques, such as intensity-modulated radiation therapy, treatment outcomes have not improved.

One of the biggest challenges in radiation therapy is that IR affects both normal tissue and solid tumours. Thus, effective radiation therapy is a way to maximise cancer cell-killing ability within acceptable ranges where adjacent healthy normal tissue can withstand radiation damage leading to improving the survival rate of HNSCC patients. Recent studies have focused on upregulation of ROS generation to induce cancer cell death or cell growth inhibition as a therapeutic strategy for increasing radiation sensitivity [23–26]. In this aspect, Nrf2, a well-studied antioxidant protein in various tumours [25, 27], is a very useful candidate to increase the ROS level. Downstream genes of Nrf2, such as CBR1, can be effective targets to regulate intracellular ROS levels in diverse tumours including HNSCC. Human CBR1 is expressed in a large variety of tissues, with high levels found in the liver, placenta, and CNS [28], consistent with a possible protective role against toxic carbonyls. CBR1 reduces highly reactive lipid aldehydes formed through oxidative stress, such as ONE, HNE, and acrolein, and catalyses a variety of endogenous and xenobiotic carbonyl compounds [7]. CBR1 affects the resistance to arsenic trioxide in leukaemia and CBR1 overexpression was sufficient to protect cells against arsenic trioxide through modulation of the generation of ROS [9]. CBR1 overexpression enhanced cell survival by decreasing oxidative stress under hypoxia, cisplatin, and doxorubicin treatment in hepatocellular carcinoma [11]. (−)-epigallocatechin gallate, a promising inhibitor of CBR1, enhanced the antitumor activity of daunorubicinol against hepatocellular carcinoma cells expressing high levels of CBR1 and corresponding xenografts [29]. There is increasing evidence that cancer cells produce higher basal levels of ROS than normal cells [30]. When endogenous oxidative stress persists, cancer cells become resistant to exogenous oxidants by enhancing endogenous antioxidant capacity [31, 32]. It is well-established that cell-killing after exposure to IR and a subset of cytotoxic chemotherapeutics is partially mediated by free radicals [33]. Therefore, we hypothesised that CBR1 could also regulate the ROS produced by IR and the inhibition of CBR1 could increase the radiation sensitivity via accumulation of ROS. First, we examined the relationship between the expression of CBR1 and IR in HNSCC cells. The mRNA and protein expression of CBR1 was increased dose-dependently by IR, suggesting that they are transcriptionally regulated by IR. The Nrf2-antioxidant DNA response element was important to maintain resistance to IR [34]. Nrf2 is known to be a transcriptional regulator of CBR1 genes under oxidative stress [10]. Consistently, our results showed that the inhibition of Nrf2 decreased the expression of CBR1 after IR. This result demonstrates that CBR1 modulates the sensitivity of radiation therapy, as a downstream molecule of Nrf2. Second, we examined whether CBR1 promotes the effect of radiation therapy for HNSCC. Our results showed that the combination of CBR1 inhibition and IR reduced colony formation in vitro and suppressed tumour growth in vivo in xenograft models. In our patient cohort, HNSCC patients with low CBR1 expression showed better survival than patients with high CBR1 expression after radiation therapy. In 174 HNSCC patients from a publicly available open database, low-expression groups of CBR1 had a better disease-free survival. These results concretely suggest that CBR1 expression can be a predictor for radiation therapy efficacy in HNSCC patients. Next, we sought to determine the mechanism by which CBR1 promotes the efficacy of radiation therapy. The suppression of CBR1 with IR resulted in IR-induced DSBs, increased ROS generation, G2-M arrest, mitotic catastrophe, and eventually a reduction in colony formation. There is a limitation in this study. HNSCC patients enrolled in this study and from publicly available database were treated with various treatment modalities rather than only radiotherapy. There are 2 purpose in the treatment for HNSCC: cure of cancer and preservation of function (breathing, speech and swallowing). Therefore, there are various treatment methods for the treatment of head and neck cancer such as concurrent chemoradiation, surgery followed by radiation, surgery followed by chemoradiation. Patients receiving only radiation are very limited such as T1, T2 larynx cancer or T1, T2 oropharynx cancer. Inevitably, it is very difficult to recruit patients with the same treatment in head and neck cancer research.

## Conclusions

Our findings suggest that CBR1 has an important role in DNA damage response through regulation of IR-mediated ROS generation causing to regulation of radiosensitivity, and CBR1 inhibition with IR might be a potent therapeutic strategy for HNSCC treatment.

## Additional files

Additional file 1: **Table S1.** Patient’s characteristics in 3 head and neck squamous cell cancer cohorts. (DOCX 18 kb)

Additional file 2: **Figure S1.** HNSCC patients with low CBR1 expression showed a good prognosis. **Figure S2.** Foci formation assay images. Representative images of the results of clonogenic survival assays. **Figure S3.** IR with CBR1 inhibition do not induce apoptosis. **Figure S4.** IR with CBR1 inhibition increases mitotic catastrophe. **Figure S5.** IR with CBR1 inhibition induce cell cycle arrest in G2/M phase. **Figure S6.** Scheme of CBR1 gene promoter-luciferase reporter constructs. **Figure S7.** Confirmation of Nrf2 mRNA expression after IR and siRNA treatment. **Figure S8.** Mouse image of Figure 6. Supplementary methods and figure legends. (ZIP 1834 kb)

## Abbreviations

CBR1: Carbonyl reductase 1
CCRT: Concurrent chemoradiotherapy
CNS: Central nervous system
DAPI: 4′,6-diamidino-2-phenylindole
DSB: Double-strand break
HNSCC: Head and neck squamous cell carcinoma
h-PP-Me: 3-(7-isopropyl-4-(methylamino)-7H-pyrrolo [2,3-d] pyrimidin-5yl) phenol
IHC: Immunohistochemistry
IR: Ionising radiation
NADPH: ᅟ
Nrf2: Nuclear factor erythroid 2–related factor 2
OS: Overall survival
ROS: Reactive oxygen species
siRNA: Small interfering RNA
TCGA: The Cancer Genome Atlas
