# Supplementary material for: Carbonyl reductase 1 is a new target to improve the effect of radiotherapy on head and neck squamous cell carcinoma
Source: J Exp Clin Cancer Res. 2018 Oct 30;37:264. doi: 10.1186/s13046-018-0942-9 (PMC6208116; doi:10.1186/s13046-018-0942-9)
Supplement: Supplementary file 1 — Table S1. Patient’s characteristics in 3 head and neck squamous cell cancer cohorts. (DOCX 18 kb) [file 13046_2018_942_MOESM1_ESM.docx]

**Table S1**. Patient’s characteristics in 3 head and neck squamous cell cancer cohorts

|  | GSE25727 | GSE42743 | GSE10300 |
| --- | --- | --- | --- |
| Number of patients | 56 | 74 | 44 |
| Gender |  |  |  |
| Male | 52 (92.9%) | 58 (78.4%) | NA |
| Female | 4 (7.1%) | 16 (21.6%) | NA |
| Age (mean ± SD) | 58.9 ± 8.9 | 58.1 ± 13.6 | NA |
| Anatomic site |  |  | NA |
| Oral cavity | 0 | 71 (95.9%) | NA |
| Oropharynx | 0 | 3 (4.1%) | NA |
| Larynx | 56(100%) | 0 | NA |
| Hypopharynx | 0 | 0 | NA |
| others | 0 | 0 | NA |
| Primary tumor |  |  |  |
| T1 | NA | 3 (4.1%) | NA |
| T2 | NA | 27 (36.5%) | NA |
| T3 | NA | 28 (37.8%) | NA |
| T4 | NA | 16 (21.6%) | NA |
| Regional lymph node |  |  |  |
| N0 | NA | NA | NA |
| N1 | NA | NA | NA |
| N2 | NA | NA | NA |
| N3 | NA | NA | NA |
| Stage |  |  |  |
| I | NA | 3 (4.1%) | NA |
| II | NA | 16 (21.6%) | NA |
| III | NA | 15 (20.3%) | NA |
| IV | NA | 40 (54.1%) | NA |
