# Supplementary material for: Carbonyl reductase 1 is a new target to improve the effect of radiotherapy on head and neck squamous cell carcinoma
Source: J Exp Clin Cancer Res. 2018 Oct 30;37:264. doi: 10.1186/s13046-018-0942-9 (PMC6208116; doi:10.1186/s13046-018-0942-9)
Supplement: Supplementary file 2 — Figure S1. HNSCC patients with low CBR1 expression showed a good prognosis. Figure S2. Foci formation assay images. Representative images of the results of clonogenic survival assays. Figure S3. IR with CBR1 inhibition do not induce apoptosis. Figure S4. IR with CBR1 inhibition increases mitotic catastrophe. Figure S5. IR with CBR1 inhibition induce cell cycle arrest in G2/M phase. Figure S6. Scheme of CBR1 gene promoter-luciferase reporter constructs. Figure S7. Confirmation of Nrf2 mRNA expression after IR and siRNA treatment. Figure S8. Mouse image of Figure 6. Supplementary methods and figure legends. (ZIP 1834 kb) [file 13046_2018_942_MOESM2_ESM.zip › Additional file 2.pptx]

## Slide 1
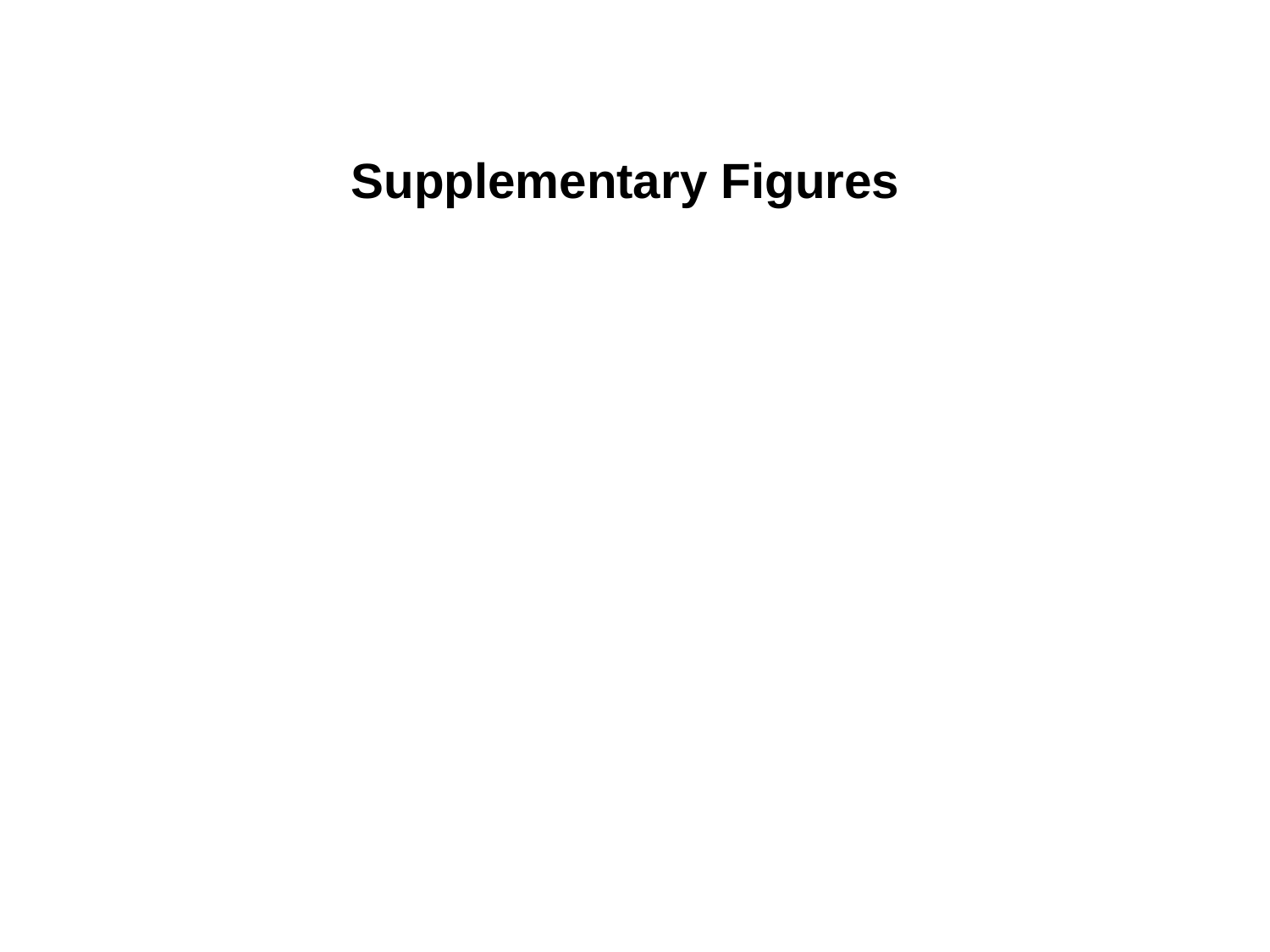

Supplementary Figures

## Slide 2
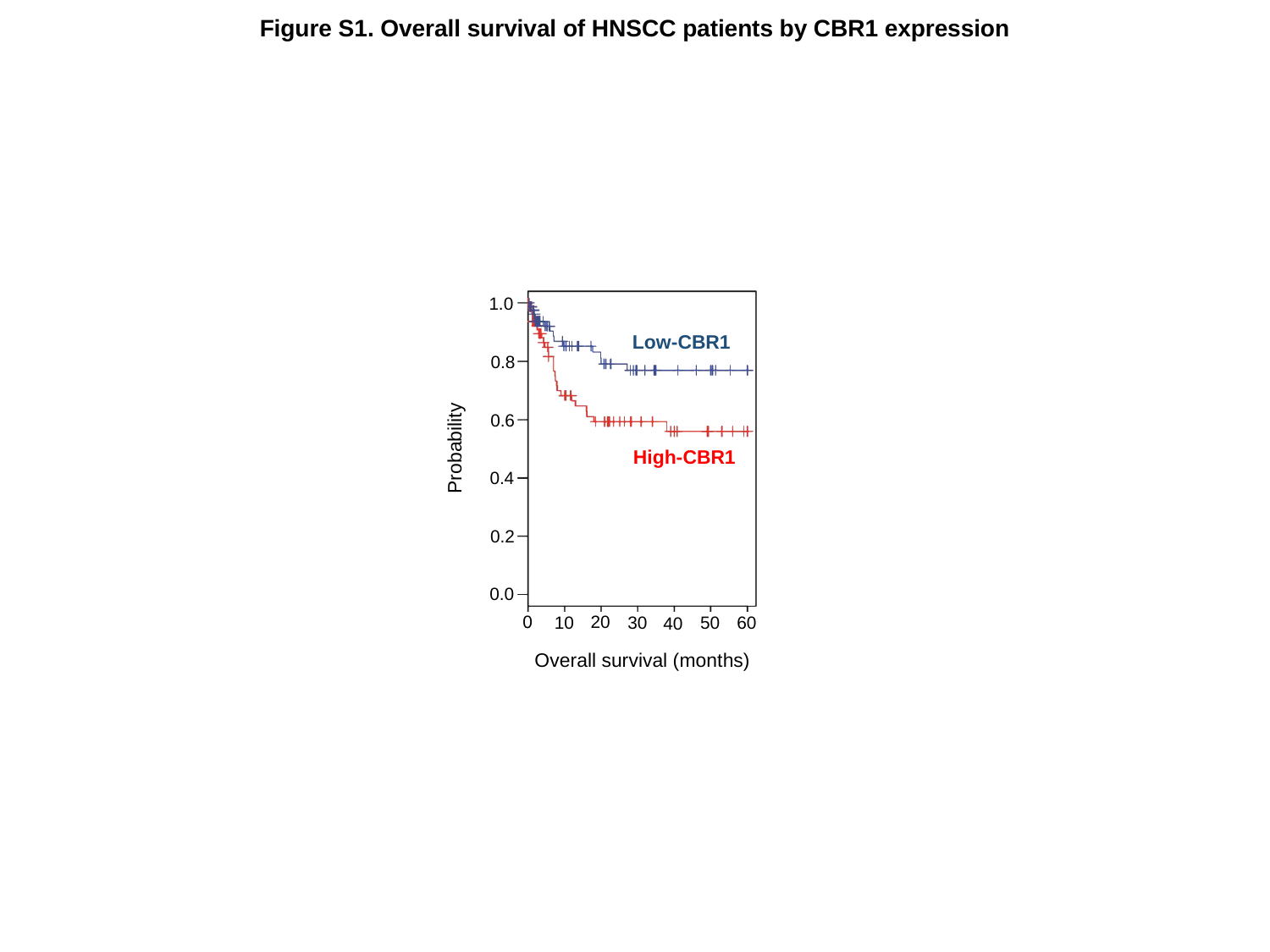

Figure S1. Overall survival of HNSCC patients by CBR1 expression
1.0
Low-CBR1
0.8
0.6
Probability
High-CBR1
0.4
0.2
0.0
0
0
20
10
50
60
30
40
Overall survival (months)

## Slide 3
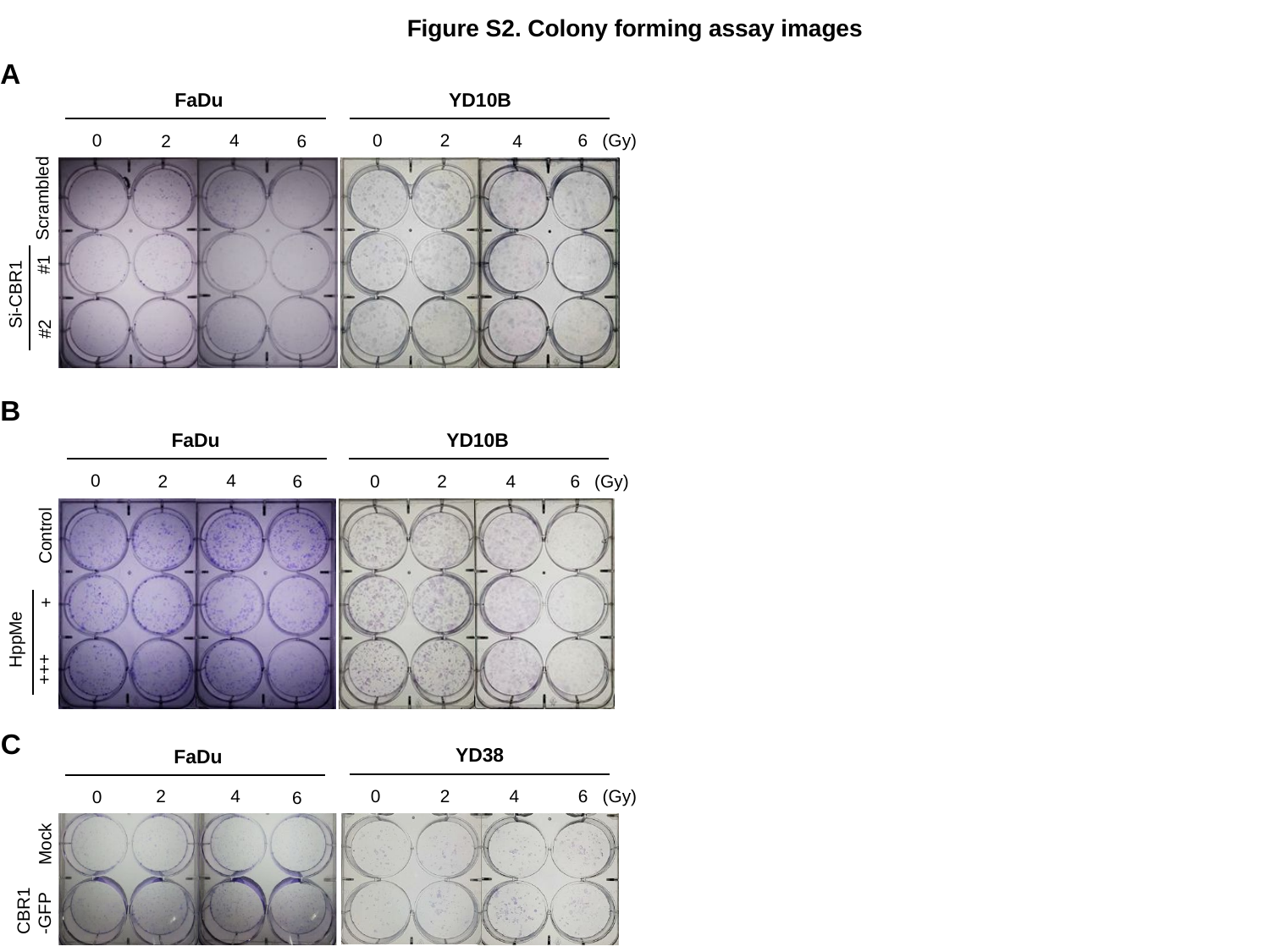

Figure S2. Colony forming assay images
A
FaDu
YD10B
0
4
0
2
6 (Gy)
4
2
6
Scrambled
#1
Si-CBR1
#2
B
FaDu
YD10B
0
4
0
6 (Gy)
2
4
6
2
Control
+
HppMe
+++
C
YD38
FaDu
2
4
0
2
4
6 (Gy)
0
6
Mock
CBR1
-GFP

## Slide 4
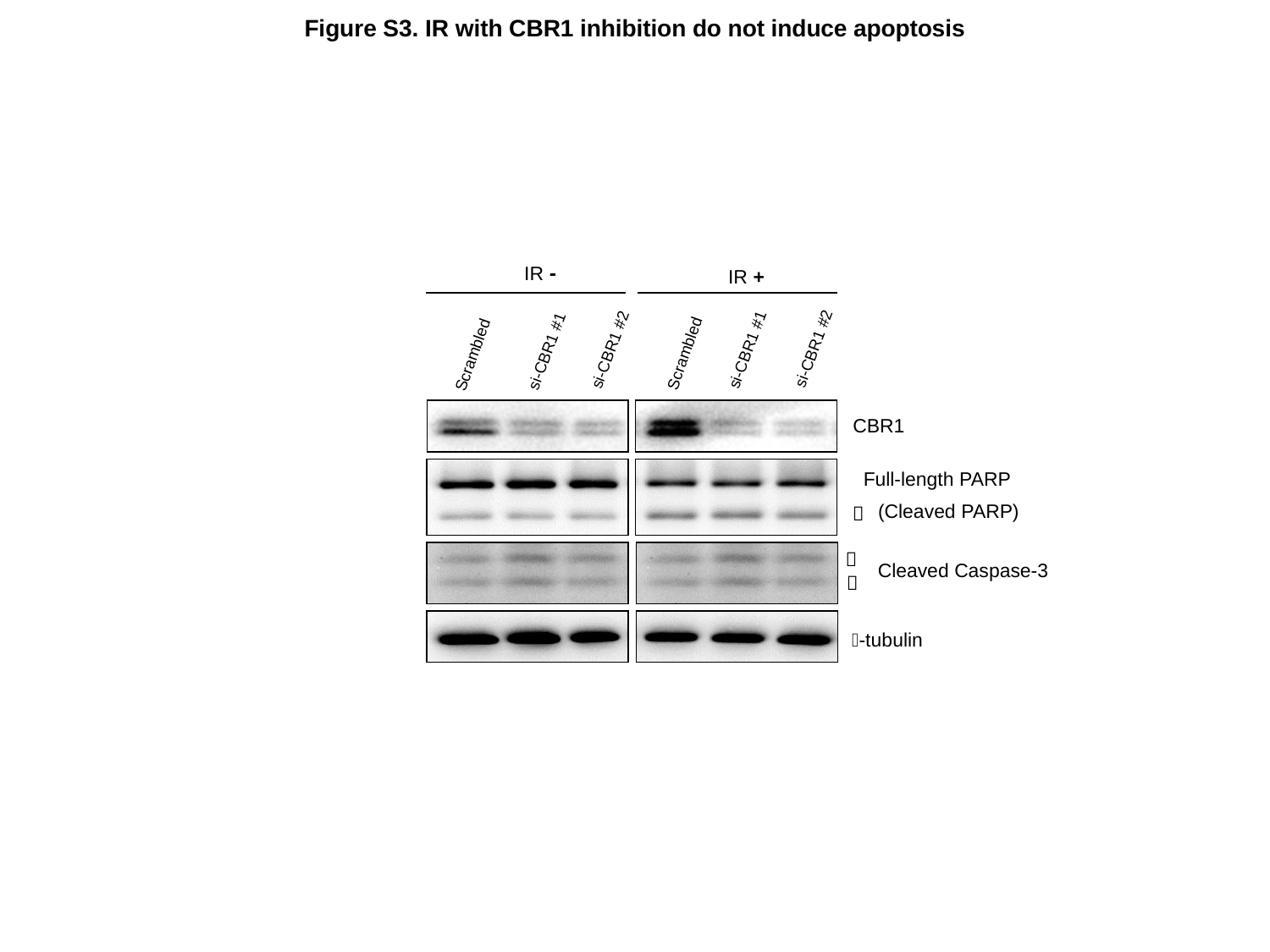

Figure S3. IR with CBR1 inhibition do not induce apoptosis
IR -
IR +
si-CBR1 #2
si-CBR1 #2
si-CBR1 #1
si-CBR1 #1
Scrambled
Scrambled
CBR1
Full-length PARP
(Cleaved PARP)


Cleaved Caspase-3

α-tubulin

## Slide 5
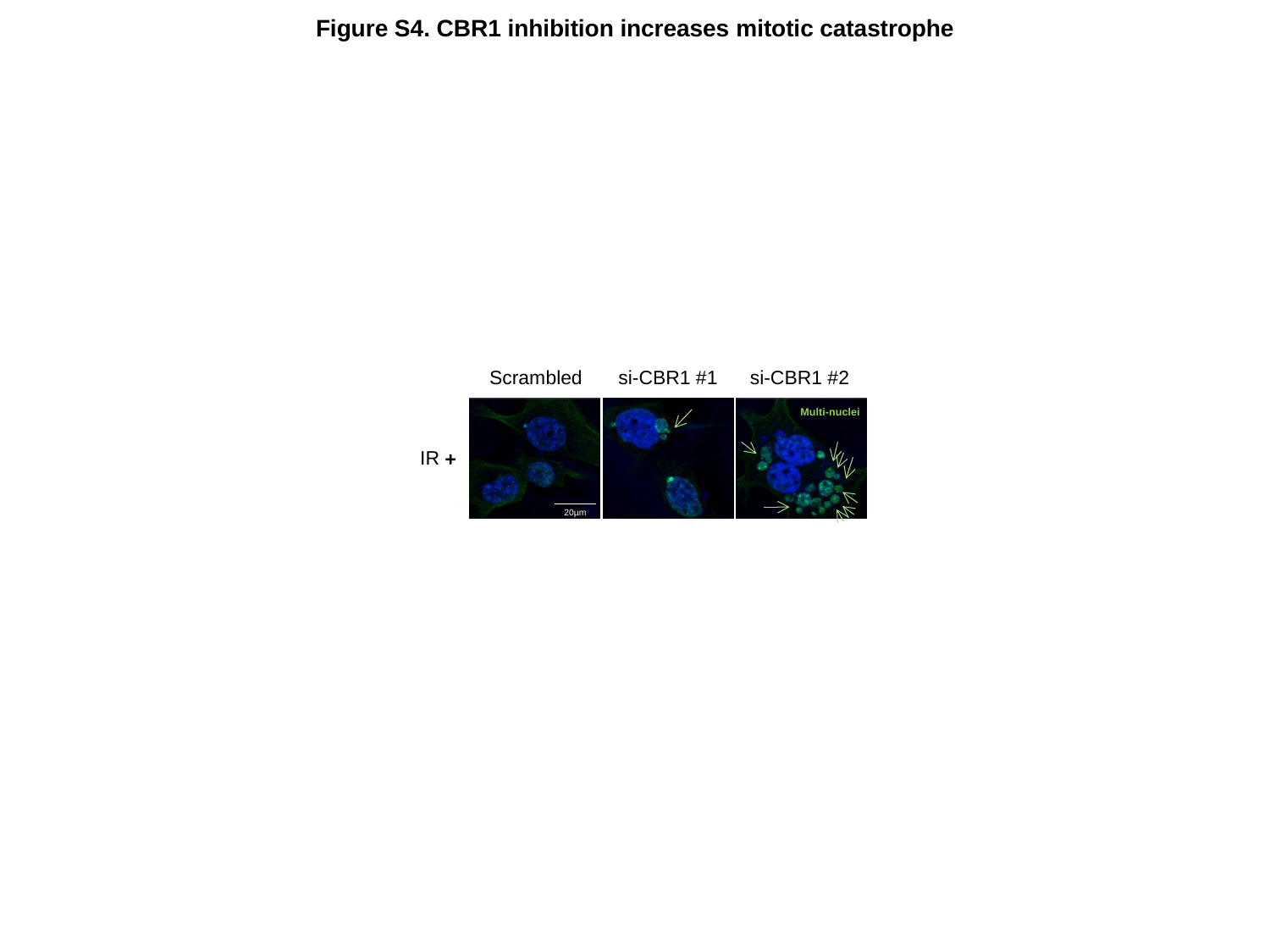

Figure S4. CBR1 inhibition increases mitotic catastrophe
Scrambled
si-CBR1 #1
si-CBR1 #2
Multi-nuclei
IR +
20µm

## Slide 6
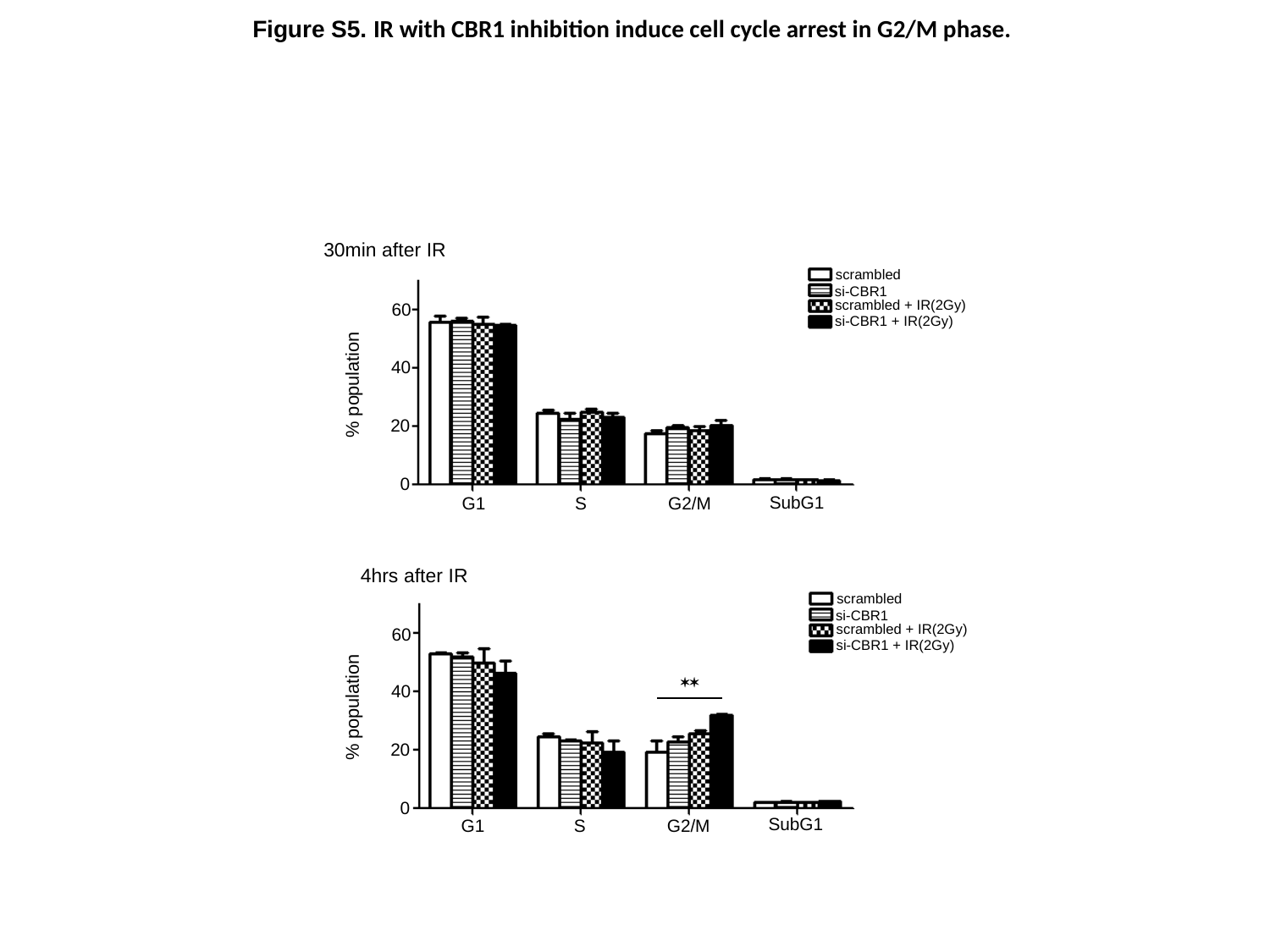

Figure S5. IR with CBR1 inhibition induce cell cycle arrest in G2/M phase.
30min after IR
scrambled
si-CBR1
scrambled + IR(2Gy)
60
si-CBR1 + IR(2Gy)
40
% population
20
0
SubG1
G1
S
G2/M
4hrs after IR
scrambled
si-CBR1
scrambled + IR(2Gy)
60
si-CBR1 + IR(2Gy)
**
40
% population
20
0
SubG1
G1
S
G2/M

## Slide 7
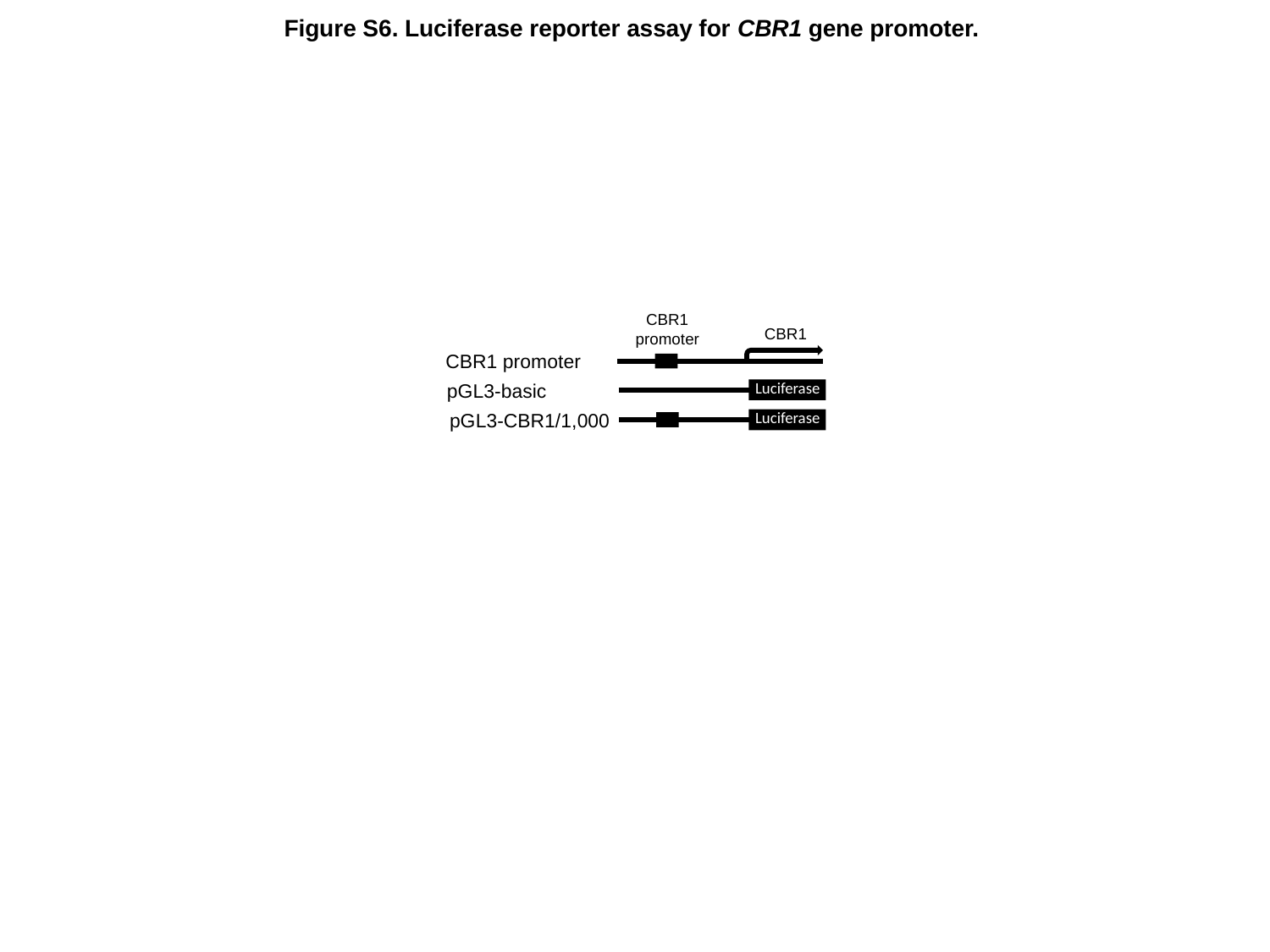

Figure S6. Luciferase reporter assay for CBR1 gene promoter.
CBR1
promoter
CBR1
CBR1 promoter
Luciferase
pGL3-basic
Luciferase
pGL3-CBR1/1,000

## Slide 8
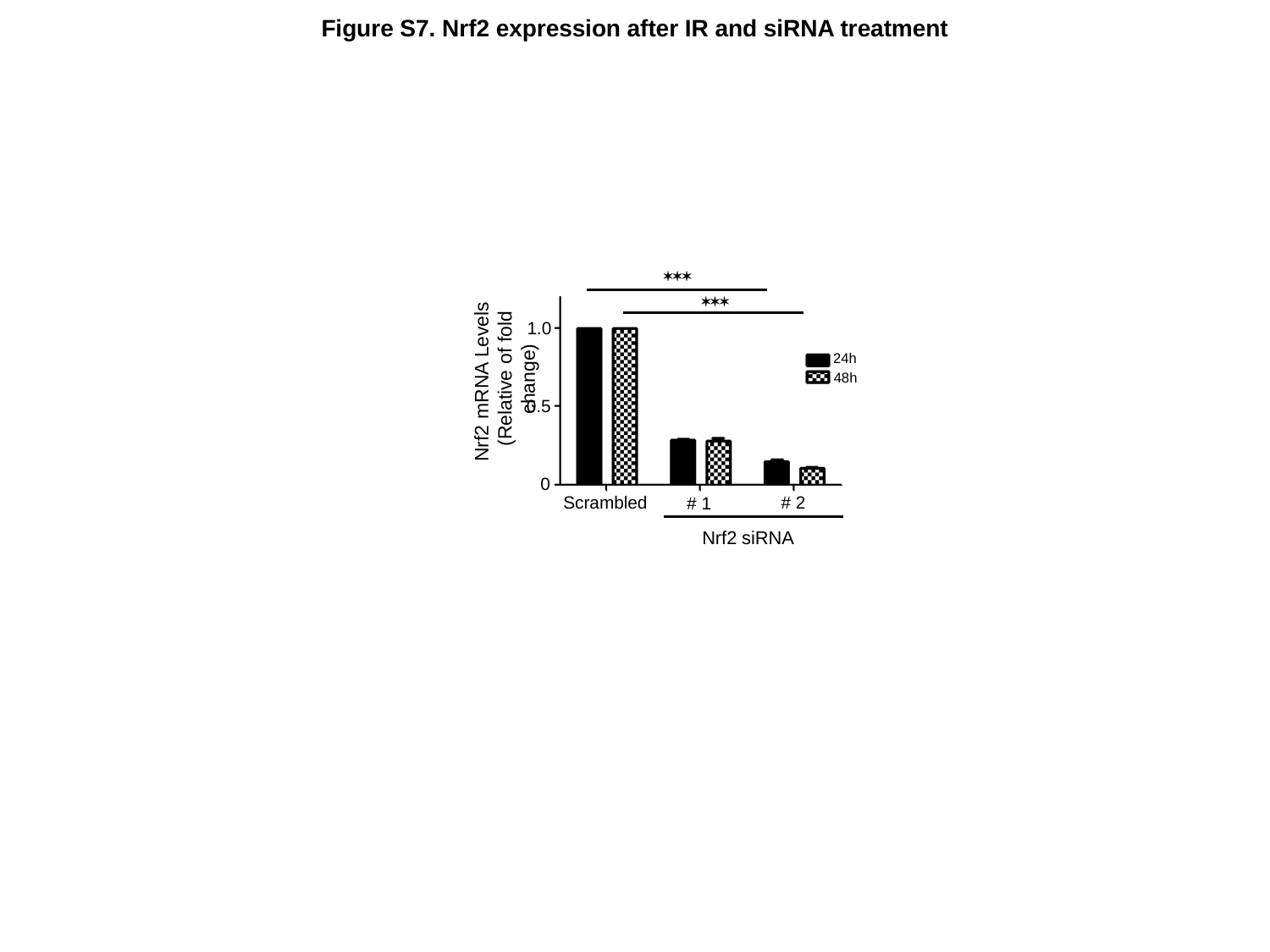

Figure S7. Nrf2 expression after IR and siRNA treatment
***
***
1.0
24h
Nrf2 mRNA Levels
(Relative of fold change)
48h
0.5
0
Scrambled
# 2
# 1
Nrf2 siRNA

## Slide 9
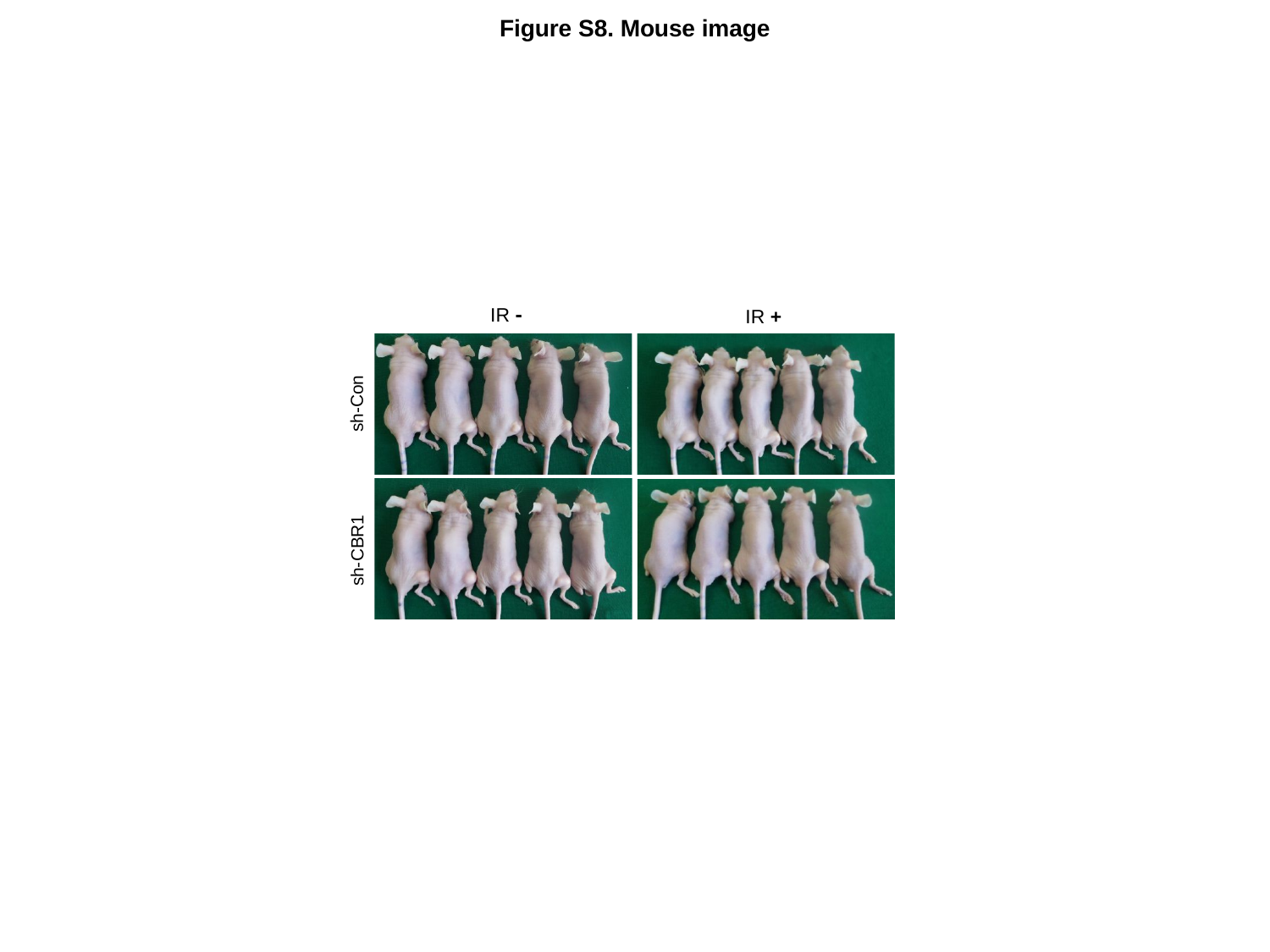

Figure S8. Mouse image
IR -
IR +
sh-Con
sh-CBR1
