# Supplementary material for: Carbonyl reductase 1 is a new target to improve the effect of radiotherapy on head and neck squamous cell carcinoma
Source: J Exp Clin Cancer Res. 2018 Oct 30;37:264. doi: 10.1186/s13046-018-0942-9 (PMC6208116; doi:10.1186/s13046-018-0942-9)
Supplement: Supplementary file 2 — Figure S1. HNSCC patients with low CBR1 expression showed a good prognosis. Figure S2. Foci formation assay images. Representative images of the results of clonogenic survival assays. Figure S3. IR with CBR1 inhibition do not induce apoptosis. Figure S4. IR with CBR1 inhibition increases mitotic catastrophe. Figure S5. IR with CBR1 inhibition induce cell cycle arrest in G2/M phase. Figure S6. Scheme of CBR1 gene promoter-luciferase reporter constructs. Figure S7. Confirmation of Nrf2 mRNA expression after IR and siRNA treatment. Figure S8. Mouse image of Figure 6. Supplementary methods and figure legends. (ZIP 1834 kb) [file 13046_2018_942_MOESM2_ESM.zip › Additional File 3.docx]

## **Supplementary Materials and Methods**

**HNSCC tissue samples and immunohistochemistry**

Eighty-five formalin-fixed and paraffin-embedded HNSCC tissues were used in this study. All patients received definite radiotherapy or adjuvant radiotherapy. Immunohistochemistry (IHC) was carried out on 4-µm tissue sections using the Bond Polymer Refine Detection System (see Supplementary Methods for tumour sections) according to the manufacturer's instructions with minor modifications. Representative paraffin blocks, selected by primary evaluation of haematoxylin-eosin stained slides, were chosen for IHC. In brief, 4-µm sections of formalin-fixed, paraffin-embedded tissues were deparaffinised with Bond Dewax Solution (LeicaBiosystems), and an antigen retrieval procedure was performed using Bond Epitope Retrieval Solution 1 (LeicaBiosystems) for 20 min at 100℃. Endogenous peroxidase was quenched by incubation with hydrogen peroxide for 15 min. Sections were incubated in a Bond-max automatic slide stainer (LeicaBiosystems) for 15 min at ambient temperature with primary polyclonal antibody for CBR1 (1:100; NBP1-86595, Novus Biologicals, Littleton, CO, USA) labelled using a biotin-free polymeric horseradish peroxidase (HRP)-linker antibody conjugate system. Bound peroxidase was visualised using a solution of diaminobenzidine as the chromogen, and nuclei were counterstained with Mayer’s haematoxylin. Stromal cells and infiltrating inflammatory cells around the tumour portion were used as an internal positive control.

**Patients and gene expression data**

We used previously published clinical and gene expression data from 158 HNSCC patients. The gene expression and clinical data of three cohorts are available from the National Center for Biotechnology Information (NCBI) Gene Expression Omnibus (GEO) database (http:// www.ncbi.nlm.nih.gov/geo). These are data from the MD Anderson Cancer Center (GSE42743, n=74) ([Lohavanichbutr *et al*, 2013](#_ENREF_24)), Vanderbilt University (GSE10300, n=44) ([Cohen *et al*, 2009](#_ENREF_6)) and Aristotle University of Thessaloniki (GSE25727, n=56) ([Fountzilas *et al*, 2012](#_ENREF_12)).

**Cell-cycle analysis**

Untreated and irradiated cells (4 Gy) were harvested by trypsinisation, washed with ice cold PBS, and fixed with 70% ethanol overnight at 20°C before DNA analysis. Following removal of ethanol by centrifugation, cells were incubated with PI Master Mix (40 mg/mL propidium iodide (Sigma-Aldrich) and 100 mg/mL DNase-free-RNase (Bioneer, Korea) in PBS) at 37°C for 30 minutes before analysis by FACSCalibur flow cytometry (BD Pharmingen, San Jose, CA, USA).

## **Supplementary Figure legends**

**Figure S1. HNSCC patients with low CBR1 expression showed a good prognosis.** Kaplan-Meier curves for overall survival based on gene expression in patients from publicly available database (n=158)

**Figure S2. Foci formation assay images.** Representative images of the results of clonogenic survival assays. A, Colony forming assay of FaDu and YD10B cells were transfected with either scrambled or CBR1 siRNA. After 24 hours, the cells were treated IR at doses of 2, 4, 6Gy. B, Colony forming assay of cells were treated with 25µM hydroxy-PP-Me (CBR1 inhibitor) and after 24 hours, the cells were treated IR at doses of 2, 4, 6Gy. C, Colony forming assay of FaDu and YD38 cells were transfected with Mock or CBR1/WT vectors and After 24 hours, the cells were treated IR at doses of 2, 4, 6Gy. 10 to 14 days after IR treatment, cells were stained with gentiana violet and representative plates are shown.

**Figure S3. IR with CBR1 inhibition do not induce apoptosis.** FaDu cells were transfected with either scrambled or CBR1 siRNA. After 24 hours, and then irradiated with 4Gy. Forty-eight hours after IR, western blot analyses were performed using antibodies against CBR1, caspase-3, PARP and α-tubulin.

**Figure S4. IR with CBR1 inhibition increases mitotic catastrophe.** Representative image and summary of the percentage of FaDu cells with micronuclei (arrows) 48hrs following treatment IR.

**Figure S5. IR with CBR1 inhibition induce cell cycle arrest in G2/M phase.** FaDu cells were transfected with scrambled or CBR1 siRNA for 24hrs and then irradiated with 0 or 2Gy. Cells were collected at each time point (30min and 4hr) thereafter analyzed by flow cytometry for the percentage of cells in G2/M (two-way ANOVA; **, P < 0.01 vs. Scrambled siRNA).

**Figure S6. Scheme of *CBR1* gene promoter-luciferase reporter constructs.** Luciferase reporter assay for CBR1 gene promoter. Detail method is described in Materials and methods of main text.

**Figure S7. Confirmation of Nrf2 mRNA expression after IR and siRNA treatment.** Nrf2 mRNA expression level was measured in FaDu cells transfected with scrambled or Nrf2-speciﬁc siRNA (two-way ANOVA; ***, P < 0.001 vs. Scrambled siRNA).

**Figure S8. Mouse image of Figure 6.** Stable shCBR1 knockdown YD10B cells (2.5×106 cells in 100 μl of saline) were injected subcutaneously in the right thigh of each mice. Radiation schedules were used during this study: 2 Gy daily for 5 days (10 Gy total). The mice were sacrificed at 39 days.
